# Chronic Thickening of the Peritoneum, Occasioning a Friction Sound

**Published:** 1860-02

**Authors:** 


*Chronic Thickening of the Peritoneum, occasioning a Friction Sound.*

Dr. Hutchinson remarked on this case as follows:—

Daniel Smith, aged 66 years, a native of Germany, a ragpicker, was admitted into the Pennsylvania Hospital, August 12th. We could learn very little of his history; but he asserted most positively that he had been sick for only six weeks, and that, previously to that time, he had been able to follow his usual occupation. His son has since told us, that twenty years ago it was thought he had phthisis, but that he entirely recovered, and has since then enjoyed good health.

At the time of his admission he was exceedingly emaciated and pale. He stated that he had become so during his illness. His pulse was always over 100, and in the evening it frequently ran up as high as 120. His skin was harsh, dry and hot. His tongue slightly coated and dryish. The percussion was abnormally clear over all parts of the chest, except between the scapulæ; on the left side it was slightly dull; on the right, more so. A mucous râle was heard at the lower part of the right lung; in all other parts the respiration was normal. There was no cough, excepting toward the close of his life. The abdomen was drawn in, but not irregularly nodu-

---

lated, as it usually is in tubercular peritonitis; it was tender to the touch, and imparted a sensation of doughiness to the hand. When the hand was passed over the abdomen, so as to cause one surface of the peritoneum to rub against the other, a grating sound was heard, and on one occasion a full respiration was sufficient to produce it; grating was also felt distinctly by the fingers. Percussion over the region of the liver indicated rather a diminution of its size. The spleen was not enlarged. He had one or two passages from his bowels daily, which were small and whitish. The urine was natural. The treatment adopted was entirely of a supporting nature. He was removed from the hospital three days before death, and died September 25th.

An autopsy was made eight hours after death. The head was not examined. *Thorax.*—Both lungs were found bound to the parietes of the chest by old adhesions, the right completely so; they were studded with large irregular masses, which were found in some cases to inclose a cretaceous substance; in the neighborhood of these nodules, and in other parts of the lungs, there was a deposit of carbonaceous matter. A similar deposit had taken place in the bronchial glands. The only healthy portion of the lungs was the lower lobe of the left lung. The heart was of normal size and presented no traces of disease, with the exception of a slight ossific degeneration of the aortic valves. *Abdomen.*—The peritoneum was found to be very much thickened, having in the median line a thickness of a quarter of an inch; on its surface were numerous minute elevations, about the size of a millet-seed. The whole of the small intestines were glued together. The liver was so adherent to the diaphragm that some force was necessary to separate it from its attachments. It was found somewhat smaller than natural, and had upon its surface bodies similar to those seen upon the peritoneum; the finger could readily be passed through its substance. The pancreas, stomach, and kidneys were healthy. There was but a small amount of effusion into the cavity of peritoneum. The deposit in the lungs, when submitted to microscopical examination, was believed to be tuberculous; its being cretaceous, and surrounded by a carbonaceous substance, gave rise to the opinion that it had been deposited twenty years before his death.

I may state, as bearing on the case, that Bennett, in his "Lectures on Clinical Medicine," says, "Nothing is more common than to see chronic tubercles surrounded by black pigmentary matter." Rokitansky also notices the association of black pigment with old tubercle.—*Proceedings of the Path. Soc. of Philadelphia.*

